# Identification of Aluminium Powder Properties for Modelling Free Air Explosions

**DOI:** 10.3390/ma15041294

**Published:** 2022-02-10

**Authors:** Piotr W. Sielicki, James Keith Clutter, Wojciech Sumelka, Tomasz Gajewski, Michał Malendowski, Piotr Peksa, Robert Studziński

**Affiliations:** 1 Faculty of Civil and Transport Engineering, Poznan University of Technology, Maria Sklodowska-Curie Street 5, 60-965 Poznan, Poland; piotr.sielicki@put.edu.pl (P.W.S.); wojciech.sumelka@put.poznan.pl (W.S.); michal.malendowski@put.poznan.pl (M.M.); piotr.peksa@put.poznan.pl (P.P.); robert.studzinski@put.poznan.pl (R.S.); 2 SciRisq, Inc., 448 W. 19th St., #210, Houston, TX 77008, USA; keith.clutter@scirisq.com

**Keywords:** aluminium powder, detonation, explosive, combustion, oxidation, equation of state

## Abstract

Aluminium is a component in many energetic formulations. Therefore, its combustion is one of the main thermochemical processes that govern the output from the energetics. Modelling aluminium combustion is a challenging task because the process is highly complex and difficult to measure. Here, tests of aluminium powder were conducted in an effort to isolate the burning of the aluminium and to determine an adequate representation of this process. Charges of 100 g and 500 g were tested, and the size of the Al/air cloud and the ratio of components in the Al/air mixture were determined, which has not been published previously. This information was used to assess the validity of the assumption that the detonation of the mixture was representative of the event. Parameters for the Jones–Wilkins–Lee equation of state for the explosive mixture and detonation products were defined. Simulations of the tests were performed, and the results were consistent with the field test data, indicating that detonation occurred when there was a mixture of 70–75% Al and 25–30% air by mass.

## 1. Introduction

The composition of an explosive is a key factor in its efficiency. Most standard military explosives and those used in research operations are condensed explosives, such as trinitrotoluene (TNT) and Plastic Explosive No. 4 (PE4). These substances are ideal for contact explosions due to their brisance and high-intensity shock wave propagation. Furthermore, they can be altered in different ways to change their explosive performances. One common alteration is the addition of various components, such as aluminium (Al) powder [1,2,3].

The addition of aluminium changes the energy release process and the overall output of an explosive at the microscopic level. Additionally, the ignition and combustion properties of an explosive can be modified, as shown by Liu et al. [4], by coating it with nanosized aluminium particles. Oxidation plays a crucial role in the combustion of aluminium. Gang et al. investigated the combustion and oxidation of Al nanoparticles at the atomistic level [5]. Depending on the amount of O_2_ and the temperature, oxidation can be grouped into three categories: mild, chain-like growth oxidation; moderate oxidation; and microexplosion-accelerated oxidation. The microexplosion-accelerated oxidation mechanism was investigated deeply by Gang et al. The burning of separated Al droplets in air was studied by Karasev et al. [6], as well as the mechanisms involved in alumina aggregate formation. 

However, the microscopic level is not the only level of interest in terms of energy release processes; the combustion of aluminium nanoparticles has also been investigated at the macroscopic level. For instance, Lewis et al. [7] studied hexahydro-1,3,5-trinitro-1,3,5-triazine (RDX) explosives with three types of aluminium nanoparticles. They showed that the addition of the nanoparticles can change the fireball temperature from 340 K to 4500 K. Gordon et al. [8] also studied the fireball of aluminised RDX, in addition to its shock wave energy. The authors reported that the shock energy is greater if aluminium is added to the high explosive rather than to the liner. Other studies of aluminised high explosives were conducted by Peuker et al. [9] and Carney et al. [10], in which optical methods were used. In the current study, the afterburn phase was analysed.

The contribution of Al combustion to the afterburn phase of aluminised explosives has been represented in various studies [11] using the Jones–Wilkins–Lee (JWL)–Miller model [12]. This model alters the energy term in the JWL equation such that it has the form

(1) $$P=A\left(1-\frac{\omega }{R_{1}V}\right)e^{-R_{1}V}+B\left(1-\frac{\omega }{R_{2}V}\right)e^{-R_{2}V}+\frac{\omega \left(E+\lambda Q\right)}{V},$$

where *Q* is the additional heat released by the aluminium particle combustion, and *λ* is the progression variable indicating the degree to which the particle has reacted. The degree of reaction equation is given by

(2) $$\frac{d\lambda }{dt}=a{\left(1-\lambda \right)}^{m}P^{n},$$

where a depends on the particle size, and m and n are reaction rate constants. This model represents the gradual addition of energy seen in the afterburn phase rather than the sudden release of energy observed in the aluminium powder explosives. Here, the rate of gasification, which is associated with the combustion rate equation, is relevant.

Other models have been introduced that also replicate the afterburn energy release due to Al combustion, but they assume that the reaction is proportional to the gasification of the particles [13]. This process is explicitly represented by the change in the diameter of the particles, which can be expressed as

(3) $$\frac{d}{dt}\left[\frac{4}{3}\pi {\left(\frac{D}{2}\right)}^{3}\rho \right]=4\pi {\left(\frac{D}{2}\right)}^{2}\dot{m_{v}},$$

which simplifies to

(4) $$D=D_{0}-\frac{2\dot{m_{v}}}{\rho }t,$$

where *D* is the particle diameter, *D*_0_ is the initial particle diameter, and *m_v_* is the gasification rate of the condensed material, which can be expressed as

(5) $$\dot{m_{v}}=\frac{\sqrt{3}}{2\pi r^{3}}\sqrt{kTm}e^{-E_{v}/RT},$$

where r is the radius of the molecule, m is the mass of each molecule, k is the Boltzmann constant (1.38 × 10^−23^ J/K), T is the temperature, $E_{v}$ is the gasification enthalpy, and R is the gas constant (8.314 J/mol·K). 

The afterburn combustion of Al can be used to redefine the degree of reaction, as follows

(6) $$\lambda =1-{\left(1-\frac{2{\dot{m}}_{v}t}{\rho_{Al}D_{0}}\right)}^{3},$$

where ${\dot{m}}_{v}$ is the gasification rate, t is time, $\rho_{Al}$ is density of aluminum, and $D_{0}$ is initial diameter.

Another group of models address the gasification of the Al particles using an empirical quasi-steady law [14]. The particle radius ($r_{p}$) rate is

(7) $$\frac{dr_{p}}{dt}=-\frac{r_{p}}{t_{b}}\left(1+0.276\sqrt{Re}\right),$$

where Re is the Reynolds number, which is based on the relative velocity between the gas and the particle, and $t_{b}$ is the burning time based on

(8) $$t_{b}=K{\left(D_{0}\right)}^{2},$$

where K is an empirical constant. According to the literature, K is typically set equal to 4 × 10^6^ s/m^2^. The mass transfer from the solid to gaseous state is

(9) $$\frac{dm_{p}}{dt}=-\frac{d}{dt}\left(\frac{4}{3}\pi \rho_{p}r_{p}^{3}\right),$$

where the subscript p denotes the particle properties. 

In the current study, the temperature of ignition depends on the state of the Al particles. If an oxide coating is present and is not cracked through some sort of physical process, then the melting point of Al_2_O_3_ would determine ignition. This melting point is approximately 2050 K. If the oxide layer is cracked, then the ignition temperature would be determined by the melting point of Al, which is approximately 950 K.

The temperature of the particles will depend on the heat feedback from the gas phase to the condensed phase. An investigation of aluminised explosives [15] has shown that the feedback can be represented using Fourier’s law

(10) $$q_{c}=\alpha_{g}\left(\frac{1}{r}\right){\left(\frac{dT}{dt}\right)}_{s},$$

where $\alpha_{g}$ is the thermal conductivity, r is the burning rate, and s denotes the gradient at the surface.

Hence, an understanding of the energy released during aluminium combustion is key to optimising the design of energetic systems and would allow the quantification of aluminium’s effect on structures. In this paper, aluminium powder was analysed at the macroscopic level; this approach enables the straightforward development of online (fast) tools that can predict the behaviour of aluminium powder explosives. The current objective was to develop an adequate representation of Al combustion to provide loading predictions for use in the analysis of the interaction between munitions and structures. From this point of view, the robust modelling of such explosives is no trivial task. 

Equations of state (EOS) are a key concept in modelling this class of energetics. Various EOS approaches are found in the literature. They have been proposed for aluminised explosive products [16], along with more popular approaches, such as the JWL formula [17,18,19]. The JWL approach is well known and has the broadest application; thus, it was adopted in the current study. Here, the expansion process of the detonation products from the composite energetics was determined to be crucial. This expansion process affects the prediction of the pressure loading at a particular distance, which is an essential characteristic in determining the effect of the explosive.

This paper analysed the combustion of Al, as this critical process affects the energy outputs of aluminised explosives. The oxidiser that was used in the tests varied according to the specifics of the energetics. In most explosives, the oxidiser is obtained from the products of the organic reaction that forms CO, CO_2_, and H_2_O. It has been shown that Al combustion is determined by the concentrations of CO_2_ and H_2_O [20]. Therefore, Al combustion often occurs before any ambient oxidiser is introduced to the Al. Here, pure Al was used to ensure that the oxidation process was related to the mixing of Al powder with ambient air. This provided insights into the combustion of Al, even when the oxidiser originated from typical detonation products.

In this study, a series of range detonation tests with pure aluminium powder mixtures were performed, which has not been published previously. The mass of the charges varied from 0.1 to 0.5 kg. The primary goal of the tests was to measure the pressure–time histories, and these results were replicated using modelling tools. Seven tests were performed, and full data recordings were obtained at standoff distances of approximately 1 and 2 m. The tests were replicated using a modelling tool that incorporated the effects of Al combustion. The EOS and reaction parameters were first defined using thermodynamic codes and analytical tools. Subsequently, as described in the final part of this paper, these values were altered to match the test results.

The paper is structured as follows. In Section 2, the test details, computational assumptions, and an explosive governing equation are presented. Section 3 identifies the explosive parameters for the 100 g charge and presents their validation for the 500 g charge. Section 4 presents the conclusions of the study.

## 2. Materials and Methods

### 2.1. Field Test Measurements

**Figure 1:**
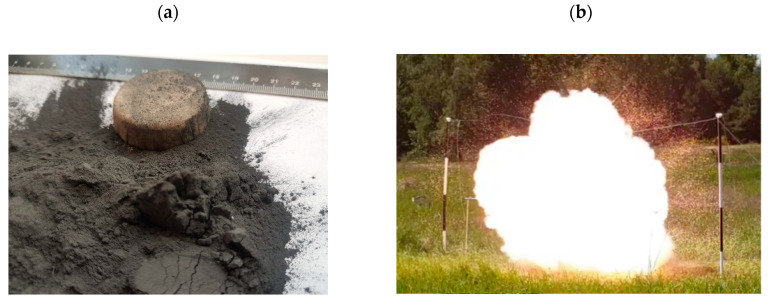

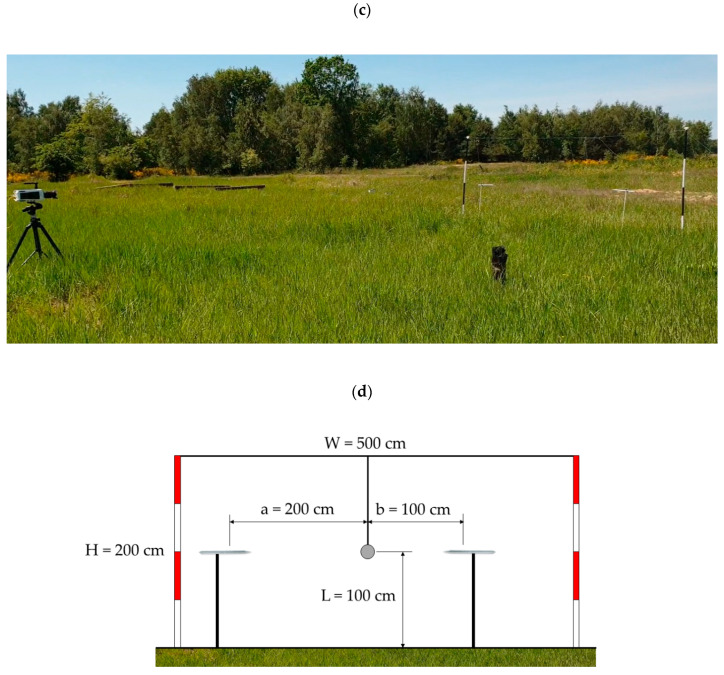
Test set up: (**a**) example of tested mixture of aluminium powder; (**b**) snapshot of the explosion; (**c**) overview of the explosion area; and (**d**) explosive devices’ mounting scheme.

In this section, data from the field tests for the 100 g and 500 g aluminium powder charge detonation are described. The experiments were conducted so as to represent a free air blast. Data from the 100 g detonation were used to determine the aluminium modelling parameters. The tests were performed using 6 µm of Al powder with an estimated density of 2.2 to 2.7 g/cc. The aluminium powder is shown in Figure 1a. Photographs from the test site are presented in Figure 1b,c, including a snapshot of the explosion (Figure 1b) and an overview of the explosion area (framed by wooden poles), along with the high-speed camera (Figure 1c). Figure 1d presents details of the explosive devices’ mounting scheme. The aluminium powder was hung in a foil bag attached to a string and was located 100 cm from the ground (L) directly between two wooden poles that were 200 cm high (H). The distance between the poles was 500 cm (W). Blast pressure pencil probes were placed at heights of 100 cm (b) and 200 cm (a) from the charge.

Seven detonations were conducted during the field tests. In the first two detonations, the aluminium powder charge mass was 100 g; in the next two, the charge mass was 200 g, and in the last three, the charge mass was 500 g. The data from the second 200 g detonation were rejected. In this case, during aluminium combustion, the foil bag was damaged, and the powder poured out of the bag. Thus, the data could not be considered for further analysis. Because the 200 g case was not represented by two detonations, this charge mass was not taken into account in the subsequent analyses. 

**Figure 2:**
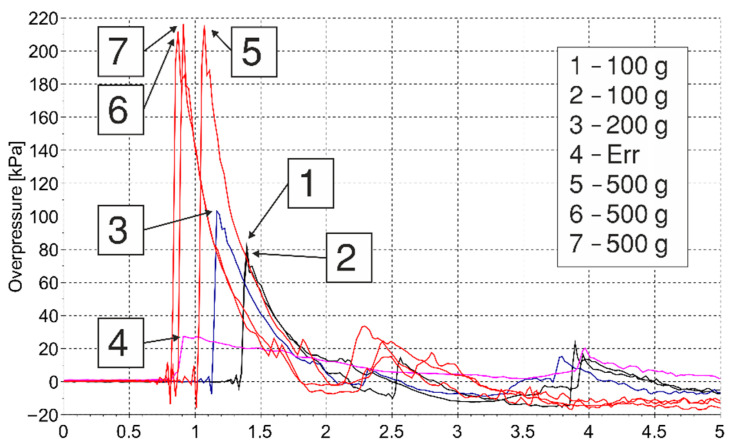
Overpressure measurements at 1 m.

**Figure 3:**
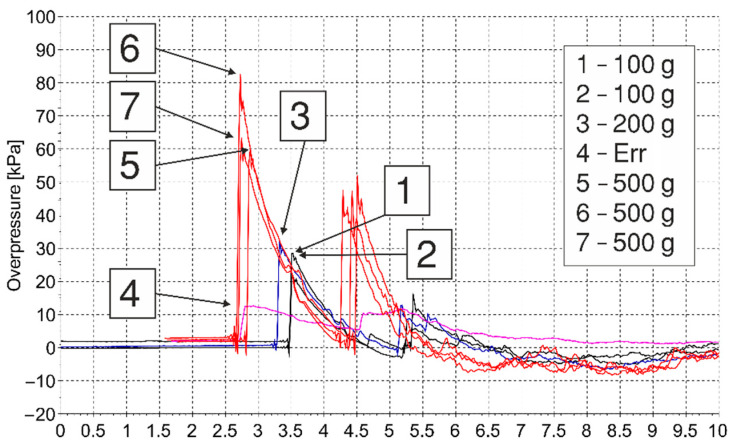
Overpressure measurements at 2 m.

The pressure–time histories from all tests were measured at 1 and 2 m from the explosive simultaneously. Two ICP^®^ blast pressure pencil probes were used simultaneously to acquire the data—these are the same probes that were used in [21]. The maximum pressure limit for these sensors is 345 kPa. The results are shown in Figure 2 and Figure 3. All measurements are presented, including those that were rejected (i.e., pressure histories no. 3 (200 g) and no. 4 (labelled as Err, i.e., error)).

**Figure 4:**
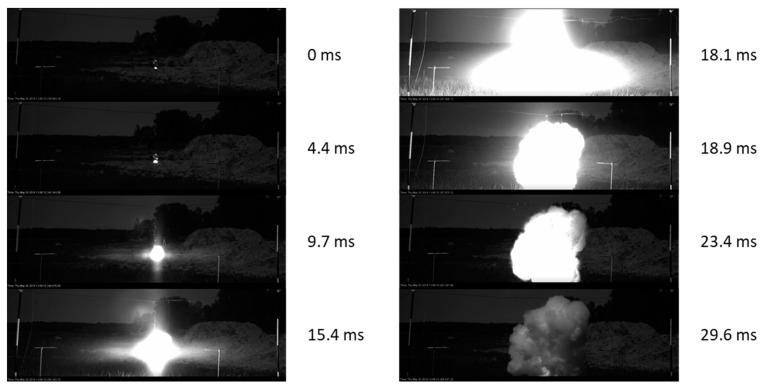
Sequence of images from the 100 g test. The time presented refers to the time after ignition.

The detonations were filmed with a high-speed camera to obtain deeper insights into the reaction process to ensure adequate modelling assumptions. In these types of experiments, a high-speed camera is typically used to assess the deformation due to the blast wave [22] and determine the speed of projectiles and fragments, e.g., [23,24]. In our tests, the high-speed camera was used to estimate the volume of the mixed powder. Figure 4 shows sample images captured immediately after detonation. The time after ignition is shown next to the sequence of images. Powder burning at the time of ignition and thereafter can be observed. 

If a spherical charge is assumed and the Al density is 2.7 g/cc, the volume of the initial charge would be 37.04 cc, and the charge would have a diameter of 2.07 cm. Notably, there was an apparent spike in output at the 18.1 ms mark. The Al/air cloud at the 18.1 ms mark had a diameter of approximately 41.4 cm and a volume of 37.15 cc. This resulted in an Al density of 2.69 × 10^−3^ g/cc. Taking the air to be at atmospheric pressure, the volume of powder and air would contain a mixture of 56% Al and 44% air considering masses. This information was used to derive an adequate representation of the explosion process.

**Figure 5:**
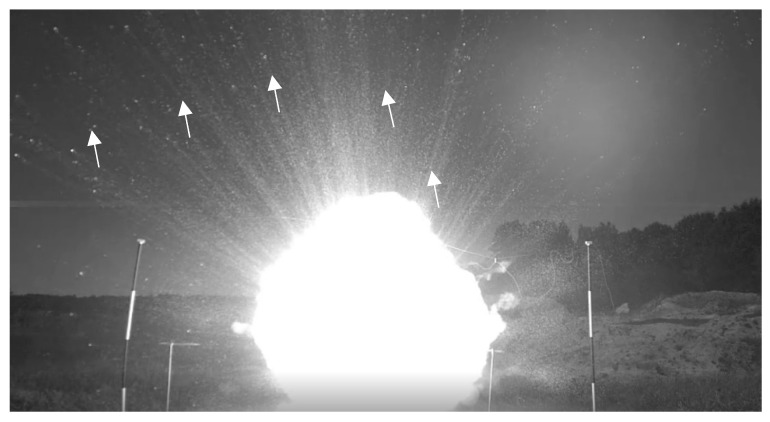
Image of the 500 g test (Test 7), in which burning Al particles (white arrows) were expelled.

The overall pattern for the 500 g tests was similar to that of the 100 g tests. For the 500 g charge, the “explosion” (rapid release of energy) appeared to occur between 2.3 and 5.8 ms after ignition. The diameter of the Al/air cloud at that time varied from 40 to 54 cm. Figure 5 shows an image taken after the “explosion” of the 500 g Al charge. More burning particles of Al were expelled outward after the “explosion” (white arrows) of the 500 g charge compared to that of the 100 g charge, suggesting that the 500 g charge was less effective than the 100 g charge. The analysis of the 500 g test data allowed an adequate representation of the Al combustion to be determined.

### 2.2. Numerical Modelling Assumptions

The computational model that was developed to simulate the energetics used a Cartesian Adaptive Mesh (CAM) framework; this model simulates scenarios involving a blast, explosion, and release of materials (thus, CAMBER). CAMBER is an object-oriented framework that utilises a variety of material models and reaction laws. The mesh adaptation was used to resolve structures under severe deformation, and the code maps out the locations of gradients in properties [25]. In CAMBER, a finite-volume formulation is used. Various flux calculation schemes have been assessed and proven to be useful in multi-material flows [26]. The advection upstream splitting method (AUSM+) scheme was selected for its simplicity and robustness [27]; high spatial order is achieved via a monotonic upstream-centred scheme for conservation law (MUSCL) extrapolation with limiters. Given the small time steps required to resolve the evolving flow field, explicit time integration was used. The code can be applied in 2D, 2D-axisymmetric, or 3D modes.

### 2.3. Governing Equation for Explosions

The objective of this study was to formulate an approach to simulate explosions involving aluminium. The energetics used in the present research were for pure aluminium, so the reaction causing the energy release was as follows

(11) $$4Al+3O\;\to \;2Al_{2}O_{3}.$$

Hence, if Al is part of the explosive composition, the oxidation could arise from the CO_2_ and H_2_O in the detonation product. In this case, the oxidiser was the O_2_ in the air. There was clearly burning Al prior to the “explosion” at 18.1 ms (see Figure 4), but at this time point, there was a sudden release of energy. It can be assumed that until this time point, the percentage of Al burned was negligible. Furthermore, the assumption was made that the explosive cloud was 56% Al and 44% air by mass (as described in Section 2.1).

A thermochemical analysis was performed as a first estimate; it was assumed that the reaction was essentially the detonation. The Chapman–Jouguet (CJ) conditions are listed in Table 1. A JWL EOS of the following form

(12) $$P=A\left(1-\frac{\omega }{R_{1}V}\right)e^{-R_{1}V}+B\left(1-\frac{\omega }{R_{2}V}\right)e^{-R_{2}V}+\frac{\omega E}{V},$$

was used for the explosive material and detonation products. The parameters used are listed in Table 2. The JWL parameters were defined for the Al/air mixture based on the behaviour of such mixtures under shock loading. The rate of the reaction was derived from the detonation velocity.

## 3. Identification and Validation

### 3.1. Identification—Simulation of 100 g Tests

**Figure 6:**
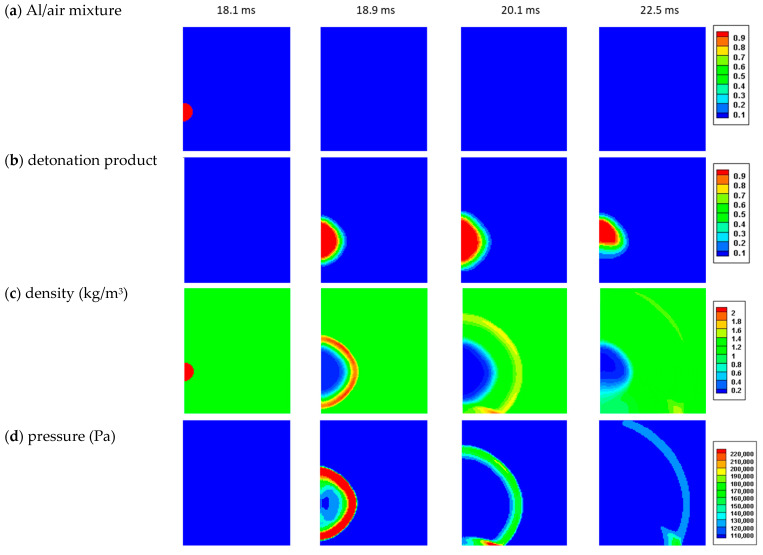
Sequence of images from the 100 g test simulation: (**a**) Al/air mixture concentration; (**b**) detonation product concentration; (**c**) density; and (**d**) pressure are shown at four time points.

**Figure 7:**
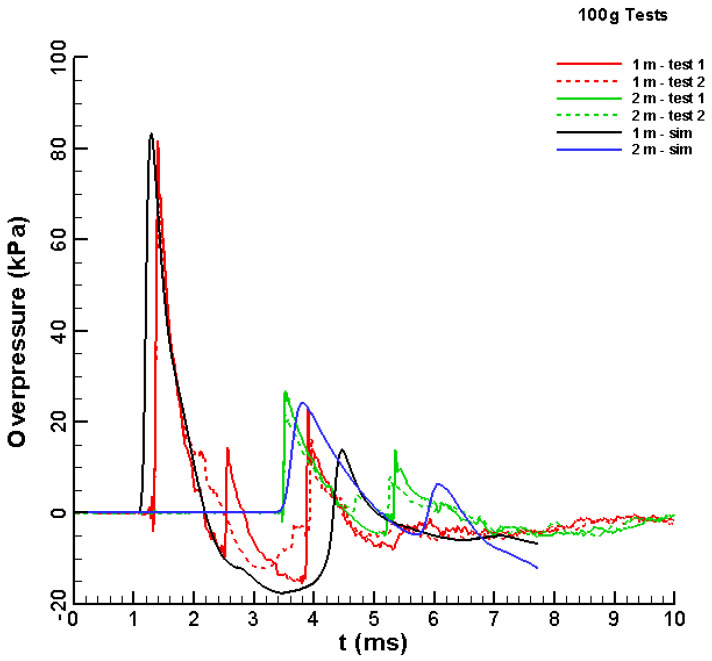
Comparisons between the test data and simulated values for the pressure versus time history at the 1- and 2 m locations for the 100 g charge.

The parameters defined in Section 2 were used to simulate the 100 g tests and identify a set of explosive parameters. The initial condition was that there was a 37.04 cc Al/air cloud. Figure 6 shows the sequence of images from the simulation. The contour maps for the concentration of reacting mixture (Al/air), the reaction products, the density, and the pressure are shown at four selected time points. The time noted is relative to t=0 for the tests. Figure 7 shows a comparison between the test data for the pressure–time histories recorded during the simulation at the 1 and 2 m locations. 

The initial pulses in pressure at both locations were similar to the test data in both magnitude and duration. The secondary pulses due to ground reflection were somewhat lower in magnitude and were delayed compared to the test data. This difference could be due to slight differences in the heights. There was an intermediate pulse in one of the 1 m test recordings. While the cause of this pulse was not clear, it was not seen in the simulation results.

These results suggest that the predominant release of energy from the combustion of the Al/air cloud can be represented as a sudden event, or, as we label it here, the “explosion”. It appears that the combustion event was essentially a mixing-controlled process. The explosion did not occur until the mass of Al powder expanded to the point where there was sufficient oxidation. Based on the size of the cloud at the time of the explosion and the mass of the Al powder, the cloud distribution was approximately 70% Al and 30% air by mass, providing an oxidiser-to-fuel mole ratio (moles O/moles Al) of 0.17, far below the stoichiometric ratio for the reaction in Equation (1).

### 3.2. Validation—Simulation of the 500 g Tests

**Figure 8:**
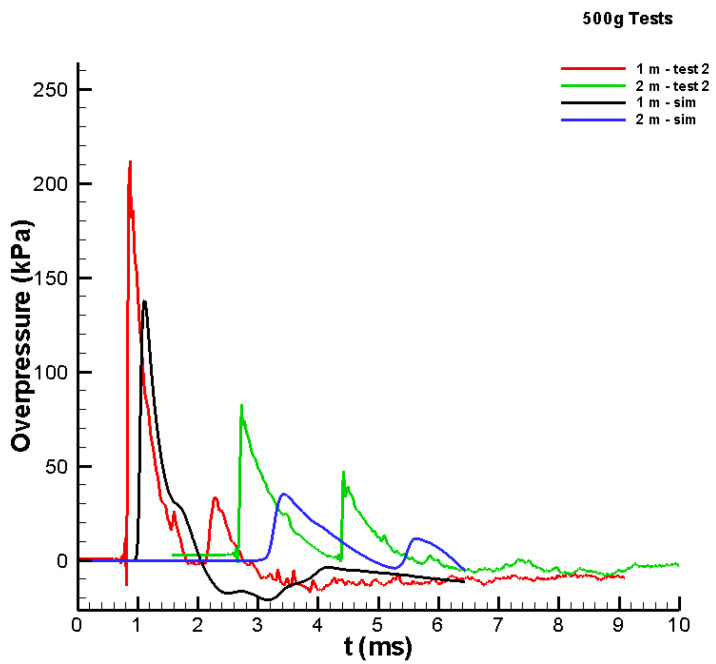
Comparison between test data and simulated values for the pressure versus time history for the 500 g Al/air cloud with a 52 cm diameter.

The 500 g tests were simulated in the validation stage. The same modelling parameters were used in terms of EOS and the rate of reaction. First, the test imagery was used to estimate the size of the spherical Al/air cloud at the point at which there was a noticeable sudden release of energy (i.e., the “explosion”). Three videos were reviewed, and the time of the explosion varied from approximately 3 to 6 ms. The size of the cloud was estimated to be approximately 52 cm in diameter. This diameter would result in a cloud composed of 84% Al and 16% air by mass. Although the fuel-to-oxidiser ratio changed slightly from the 100 g case, the same parameters used in that case were applied. The pressure–time histories predicted at the 1 and 2 m locations are shown in Figure 8. At both locations, the peak simulated pressures were lower than the peak measured pressures.

**Figure 9:**
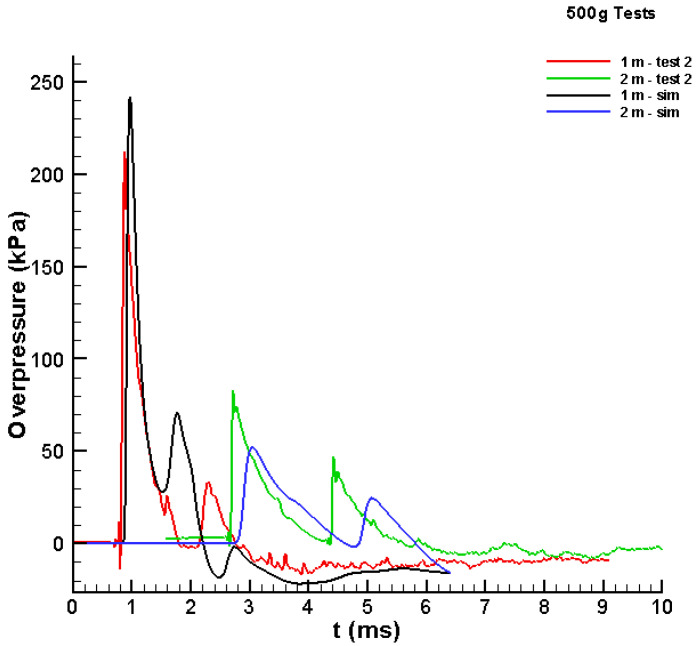
Comparison between test data and simulated values for the pressure versus time history for the 500 g Al/air cloud with a 63 cm diameter.

Because the cloud estimated from the video produced a lower output, several cloud sizes were applied until a good match between the measured and simulated pressure–time histories was achieved. Figure 9 shows the results when the diameter of the Al/air cloud was assumed to be 63 cm. The comparison is moderately good, wherein the peaks at the 2 m location are slightly lower than those in the test data. Using the assumed size of the cloud and the mass of Al, the cloud was found to be 75% Al and 25% air by mass. This result was similar to the ratio found for the 100 g results. The study presented shows that combining the experimental and advanced numerical approaches creates the ability to obtain the synergic effect for better understanding the physical phenomena, similar to [28].

## 4. Conclusions

Combustion is the key process in many energetic systems. In this paper, the combustion of Al was investigated using a series of tests and simulations, which has not been published previously in the literature. Here, the Al was isolated and allowed to react with just oxygen in ambient air; the oxidation arose from the detonation products. The results of the 100 g and 500 g Al charges indicated that the sudden explosions occurred when a mixture of 70–75% Al and 30–25% air, by mass, was obtained.

Among others, the novelty of the paper is that the output from the explosions was replicated using the JWL state equation for the product and assuming detonation of the mixture. The detonation was modelled using a prescriptive method that set the burn rate based on the detonation velocity. The delay from the initiation of the event until the time of the explosion is currently under investigation. Further studies will aim to determine whether this delay is related to the time required for the gasification of the Al or the event is a mixing-controlled process.

## Figures and Tables

**Table 1:** Detonation conditions assumed in the computational thermochemical code for the Al/air explosion.

P (GPa)	UCJ (km/s)	V (cc/g)	T (K)	C (km/s)	γ
0.002	1.166	192.557	3108.4	0.602	0.954

**Table 2:** JWL parameters assumed in the computational thermochemical code for the detonation products.

Material	ρ_o_ (g/cc)	A (GPa)	B (GPa)	R_1_	R_2_	ω	E_o_ (kJ/cc)
explosive	2.68	1.43 × 10^−1^	−5.6 × 10^−4^	21.875	0.33	0.3507	0
detonation products	2.68	2.86 × 10^−2^	2.8 × 10^−3^	7.0	0.50	0.3507	2.4 × 10^−3^ *

* This value was adjusted to 2.7 × 10^−3^ after initial simulations of the 100 g case to better match the test data.

## Data Availability

The data presented in this study are available on request from the corresponding author.
